# Muscle MRI in patients with dysferlinopathy: pattern recognition and implications for clinical trials

**DOI:** 10.1136/jnnp-2017-317488

**Published:** 2018-05-07

**Authors:** Jordi Diaz-Manera, Roberto Fernandez-Torron, Jaume LLauger, Meredith K James, Anna Mayhew, Fiona E Smith, Ursula R Moore, Andrew M Blamire, Pierre G Carlier, Laura Rufibach, Plavi Mittal, Michelle Eagle, Marni Jacobs, Tim Hodgson, Dorothy Wallace, Louise Ward, Mark Smith, Roberto Stramare, Alessandro Rampado, Noriko Sato, Takeshi Tamaru, Bruce Harwick, Susana Rico Gala, Suna Turk, Eva M Coppenrath, Glenn Foster, David Bendahan, Yann Le Fur, Stanley T Fricke, Hansel Otero, Sheryl L Foster, Anthony Peduto, Anne Marie Sawyer, Heather Hilsden, Hanns Lochmuller, Ulrike Grieben, Simone Spuler, Carolina Tesi Rocha, John W Day, Kristi J Jones, Diana X Bharucha-Goebel, Emmanuelle Salort-Campana, Matthew Harms, Alan Pestronk, Sabine Krause, Olivia Schreiber-Katz, Maggie C Walter, Carmen Paradas, Jean-Yves Hogrel, Tanya Stojkovic, Shin’ichi Takeda, Madoka Mori-Yoshimura, Elena Bravver, Susan Sparks, Luca Bello, Claudio Semplicini, Elena Pegoraro, Jerry R Mendell, Kate Bushby, Volker Straub, The Jain COS Consortium, Adrienne Arrieta

**Affiliations:** 1 Centro de Investigación Biomédica en Red en Enfermedades Raras (CIBERER), Barcelona, Spain; 2 Neuromuscular Disorders Unit, Neurology Department, Hospital de la Santa Creu i Sant Pau, Barcelona, Spain; 3 Neuromuscular Area, Biodonostia Health Research Institute, Neurology Service, Donostia University Hospital, Donostia-San Sebastian, Spain; 4 The John Walton Muscular Dystrophy Research Centre, MRC Centre for Neuromuscular Diseases, Newcastle upon Tyne, UK; 5 Radiology Department, Universitat Autònoma de Barcelona, Hospital de la Santa Creu i Sant Pau, Barcelona, Spain; 6 Magnetic Resonance Centre, Institute for Cellular Medicine, Newcastle University, Newcastle upon Tyne, UK; 7 AIM & CEA NMR Laboratory, Institute of Myology, Pitié-Salpêtrière University Hospital, Paris, France; 8 The Jain Foundation, Seattle, Washington, USA; 9 Center for Translational Science, Division of Biostatistics and Study Methodology, Children’s National Health System, Washington, District of Columbia, USA; 10 Department of Pediatrics, Epidemiology and Biostatistics, George Washington University, Washington, District of Columbia, USA; 11 Department of Radiology, Nationwide Children’s Hospital, Columbus, Ohio, USA; 12 Radiology Unit, Department of Medicine, University of Padova, Padova, Italy; 13 Department of Radiology, National Center Hospital, National Center of Neurology and Psychiatry, Tokyo, Japan; 14 Department of Radiology, CMC Mercy Charlotte, Carolinas Healthcare System Neurosciences Institute, Charlotte, North Carolina, USA; 15 Department of Radiology, Hospital U. Virgen de Valme, Sevilla, Spain; 16 Department of Clinical Radiology, Ludwig-Maximilians-University, Munich, Germany; 17 Center for Clinical Imaging Research CCIR, Washington University, St. Louis, Missouri, USA; 18 Centre de Résonance, Magnétique Biologique et Médicale, Marseille, France; 19 Aix-Marseille Université, Marseille, France; 20 Department of Diagnostic Imaging and Radiology, Children’s National Health System, Washington, District of Columbia, USA; 21 Department of Radiology, Westmead Hospital, Westmead, New South Wales, Australia; 22 Faculty of Health Sciences, University of Sydney, Sydney, Australia; 23 Lucas Center for Imaging, Stanford University School of Medicine, Stanford, California, USA; 24 Charite Muscle Research Unit, Experimental and Clinical Research Center, A Joint Co-operation of the Charité Medical Faculty and the Max Delbrück Center for Molecular Medicine, Berlin, Germany; 25 Department of Neurology and Neurological Sciences, Stanford University School of Medicine, Stanford, California, USA; 26 Institute for Neuroscience and Muscle Research, Children’s Hospital at Westmead, University of Sydney, Sydney, New South Wales, Australia; 27 Department of Neurology, Children’s National Health System, Washington, District of Columbia, USA; 28 National Institutes of Health (NINDS), Bethesda, Maryland, USA; 29 Neuromuscular and ALS Center, La Timone Hospital, Aix-Marseille Université, Marseille, France; 30 Department of Neurology, Washington University School of Medicine, St. Louis, Missouri, USA; 31 Friedrich-Baur-Institute, Department of Neurology, Ludwig-Maximilians-University of Munich, Munich, Germany; 32 Neuromuscular Unit, Department of Neurology, Hospital U. Virgen del Rocío/Instituto de Biomedicina de Sevilla, Sevilla, Spain; 33 Institut de Myologie, AP-HP, G.H. Pitié-Salpêtrière, Paris, Île-de-France, France; 34 Department of Neurology, National Center Hospital, National Center of Neurology and Psychiatry, Kodaira, Tokyo, Japan; 35 Neurosciences Institute, Carolinas Healthcare System, Charlotte, North Carolina, USA; 36 Department of Neurosciences, University of Padova, Padova, Italy; 37 Nationwide Children’s Hospital, Columbus, Ohio, USA

**Keywords:** muscle MRI, muscular dystrophy, dysferlinopathy, outcome measures

## Abstract

**Background and objective:**

Dysferlinopathies are a group of muscle disorders caused by mutations in the *DYSF* gene. Previous muscle imaging studies describe a selective pattern of muscle involvement in smaller patient cohorts, but a large imaging study across the entire spectrum of the dysferlinopathies had not been performed and previous imaging findings were not correlated with functional tests.

**Methods:**

We present cross-sectional T1-weighted muscle MRI data from 182 patients with genetically confirmed dysferlinopathies. We have analysed the pattern of muscles involved in the disease using hierarchical analysis and presented it as heatmaps. Results of the MRI scans have been correlated with relevant functional tests for each region of the body analysed.

**Results:**

In 181 of the 182 patients scanned, we observed muscle pathology on T1-weighted images, with the *gastrocnemius medialis* and the *soleus* being the most commonly affected muscles. A similar pattern of involvement was identified in most patients regardless of their clinical presentation. Increased muscle pathology on MRI correlated positively with disease duration and functional impairment.

**Conclusions:**

The information generated by this study is of high diagnostic value and important for clinical trial development. We have been able to describe a pattern that can be considered as characteristic of dysferlinopathy. We have defined the natural history of the disease from a radiological point of view. These results enabled the identification of the most relevant regions of interest for quantitative MRI in longitudinal studies, such as clinical trials.

**Clinical trial registration:**

NCT01676077.

## Introduction

Dysferlinopathies are a group of autosomal recessive muscular dystrophies caused by mutations in the *DYSF* gene.^1 2^ The absence or deficiency of dysferlin leads to muscle fibre necrosis and replacement by fat and fibrous tissue. The two most frequent presentations are limb girdle muscular dystrophy type 2B (LGMD-2B) and distal myopathy with calf involvement or Miyoshi myopathy (MM).^3^ Other phenotypes, such as distal myopathy with anterior tibial involvement, proximodistal weakness and pseudometabolic presentation, have also been described.^4^ Clinical symptoms usually start in young adulthood and are associated with highly elevated serum creatine kinase levels. The disease progresses invariably producing muscle weakness that markedly impairs daily life activities. Respiratory and cardiac involvement is uncommon in patients with dysferlinopathy.^5^

In recent years, muscle MRI protocols have been developed to assess disease progression in muscular dystrophies using sequences that are able to quantify the amount of fat replacement per muscle.^6 7^ Previous muscle MRI studies in dysferlinopathy showed initial involvement of the *adductor magnus*, *semimembranosus* and *vastus lateralis* muscles in the thigh and *gastrocnemius medialis*, *soleus* and *tibialis anterior* in the legs. The pattern of muscle involvement has not been described to vary between phenotypes, although these findings were reported in small cohorts.^8–11^

Natural history studies are essential to understand the progressive course of muscular dystrophies and to identify suitable outcome measures that can be used in future clinical trials. To address this gap, the Jain Foundation is funding The Clinical Outcome Study for Dysferlinopathy, a multicentre natural history study in a large cohort of patients (http://www.jain-foundation.org).^12^ Our aims in this paper are to identify the pattern of muscle pathology using MRI in a large cohort of patients with dysferlinopathy, to identify which muscles might be suitable to assess by quantitative MRI and study how MRI findings correlate with functional tests.

## Methods

### Study set-up and subjects

Two hundred and one patients with dysferlinopathy from 15 sites (Newcastle, Barcelona, Seville, Munich, Berlin, Padua, Marseille, Paris, Saint Louis, Columbus, Charlotte, Washington DC, Stanford, Tokyo and Sydney) were enrolled in the study. One hundred and eighty-two patients had an MRI scan (clinical and genetic details are described in online supplementary file 1). Inclusion criteria were ≥2 pathogenic mutations in *DYSF* or 1 pathogenic mutation plus either absent dysferlin expression on skeletal muscle immunoblot or ≤20% blood dysferlin monocyte expression. Truncating mutations and splice site mutations affecting the +1/–1 or +2/–2 positions were deemed pathogenic. Pathogenicity of other splice site mutations and missense mutations was defined according to the UMD Predictor (http://umd-predictor.eu).

10.1136/jnnp-2017-317488.supp1 Supplementary file 1

Demographic data were collected for ethnicity, gender, age, ambulatory status and disease duration. Patients were stratified according to the pattern of weakness at disease onset: (1) LGMD-2B, (2) MM, (3) proximodistal weakness, (4) other (pseudometabolic weakness). Those patients with no weakness at baseline examination but hyperCKemia were considered as (5) asymptomatic hyperCKemia. Disease duration was based on the time from onset of muscle weakness in symptomatic patients and time from first abnormal blood analysis result in patients with isolated hyperCKemia.

### Functional status and physiotherapy assessment

Ambulation status was determined by the ability to walk 10 m with shoes and usual walking aids or orthotics. Physiotherapists assessed muscle strength in upper and lower limbs by manual muscle testing (MMT) using an Medical Research Council 11-point scale (0=no movement, 1, 2, 3–, 3, 3+, 4–, 4, 4+, 5–, 5=no weakness). A dysferlinopathy-adapted 22-item scale based on the original 17-item North Star Ambulatory Assessment (NSAA) scale used in Duchenne muscular dystrophy and the Motor Function Measure were used to assess motor performance.^12 13^ Timed tests (6 min walk test, rise from floor, 10 m walk/run, time to climb and descend four steps and timed up and go tests) were performed in ambulant patients. The Brooke scale was performed to evaluate upper limb functional status and the ACTIVLIM as a patient-reported outcome measure.^14 15^

### Muscle MRI: acquisition

One hundred and eighty-two patients underwent a baseline muscle MRI scan, of which 84 patients had whole body and 98 patients lower limb scans. The core protocol consisted of T1-weighted, Dixon, B1 map and T2-weighted sequences. Here, we report the findings using anatomical T1-weighted sequences. The manufacturer, models and axial T1-weighted parameters are detailed in online supplementary file 2. The quality of the MRI studies was analysed by the study radiologists.

10.1136/jnnp-2017-317488.supp2 Supplementary file 2

### Muscle MRI: Semiquantitative assessment

A blinded neurologist (RF-T) and radiologist (JL), both with experience in muscle MRI in neuromuscular disorders, independently evaluated axial T1-weighted sequences with the semiquantitative Mercuri visual scale, modified by Fischer, described in online supplementary file 2.^16^ The observers agreed on the scoring of 96% of muscles. Inter-rater agreement kappa was 0.93 (95% CI 0.91 to 0.96). For the remaining 4%, observers reviewed the muscles together and agreed a final score.

### Genotype-MRI correlation

We divided the cohort into two groups depending on mutation type: (1) patients in whom absent dysferlin expression was predicted (harbouring homozygous or compound heterozygous truncating mutations) and (2) patients in whom reduced dysferlin expression was predicted (two missense mutations or one missense mutation and one truncating mutation). We compared median value of muscle fatty replacement between groups using the Wilcoxon-Mann-Whitney test.

### Statistics

We used the Shapiro-Wilk test to confirm that none of our variables were normally distributed. As such, non-parametric statistic tests were used for the analysis.

The Mann-Whitney U test was used to compare quantitative variables and the χ² test to compare qualitative variables. Due to the high number of comparisons studied, Bonferroni correction was used as posthoc analysis. To investigate correlations between muscle function tests and MRI findings, Spearman’s rank correlation was used (coefficient reported as ρ). The correlation was considered significant if p value was less than 0.05 and ρ was 0.6 or higher. Hierarchical analysis and graphical representation as a heatmap was performed using R software, V.3.1.3. Statistical analyses were performed using IBM SPSS Statistics, V.21 (IBM, Armonk, New York, USA).

### Standard protocol approvals, registrations and patient consents

All participants provided informed consent. The study was approved by ethical review boards at each centre and registered at ClinicalTrials.gov (NCT01676077).

## Results

### Patients

We included 182 patients (91 women, mean age at MRI 38±12.6 years) from whom we have obtained muscle MRI scans. Demographic, clinical and genetic data are summarised in online supplementary file 3.

10.1136/jnnp-2017-317488.supp3 Supplementary file 3

### Muscle MRI involvement: general features

Signal abnormalities in T1-weighted images were detected in all but one patient (181 out of 182 patients). Asymmetric involvement, judged as a score difference of at least 2 points in at least one muscle, was found in 77 patients (41.8%) although in 51/77 patients the asymmetry was found in one muscle only. Asymmetry in two or more muscles was found in 26/77 patients. Muscles that had asymmetric involvement were variable.

### Cranial muscles

**Figure 1:**
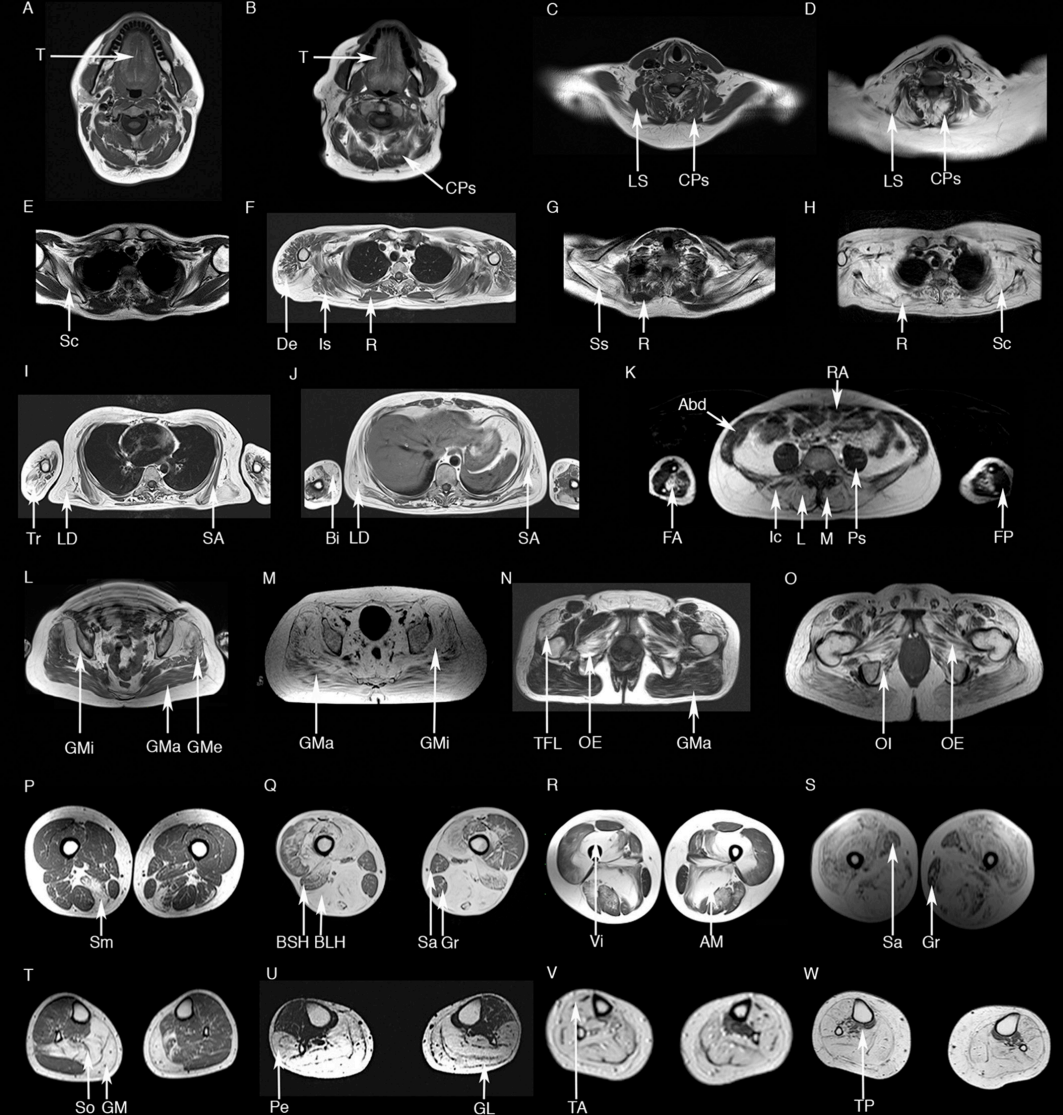
Axial T1-weighted muscle MRI in dysferlinopathy. The cranial muscles (A–B) more commonly replaced by fat are the tongue (T) and the cervical paraspinal muscles (CPs). *Levator scapulae* (LS) is generally not involved until later stages of the disease (C, no involvement; D, involvement). In the scapular region (E–H) the *subscapularis* (Sc) is involved at the early stages; other muscles such as *deltoid* (De), *infraspinatus* (Is) or *supraspinatus* (SS) become progressively involved. *Rhomboideus* (R) tends to be not involved until later stages of the disease (E–G, no involvement; H, involvement in an advanced case). *Biceps brachii* (Bi), *triceps brachii* (Tr) and the anterior muscles of the forearm (FA) are commonly involved (I–K), while the posterior muscles of the forearm (FP) are not involved even in later stages of the disease. *Latissimus dorsi* (LD) tends to be involved before *serratus anterior* (SA) in most of the patients (I–J). Paraspinal muscles including the *multifidus* (M), the *longissimus* (L) and the *iliocostalis* (Ic) are affected in most of the patients at symptom onset, while abdominal muscles, such as *rectus abdominis* (RA) are more resistant and become affected only in latter stages (K). *Gluteus minor* (GMi) is more severely involved than *gluteus medius* (GMe) and *maximus* (GMa) (L–M). Pelvic floor muscles are transformed by fat in patients with dysferlinopathy, with the *tensor fascia latae* (TFL), *obturatorius externus* (OE) and *internus* (OI) being the muscles more commonly involved (N–O). The posterior muscles of the thighs (*semimembranosus* (Sm), *biceps femoris long head* (BLH), *biceps femoris short head* (BSH) or *adductor major* (AM)) are commonly involved in most of the patients (P–S). BSH tend to be less involved than BLH in early and mid-stage patients (Q). *Vasti* muscles are commonly involved even in early stages of the diseases (*vastus intermedius* (VI) in R). In contrast *Sartorius* (Sa) and *gracilis* (Gr) are not involved until late stages of the disease (Q and S). Analysis of the lower legs (T–W) shows initial involvement of *gastrocnemius medialis* (GM) and *lateralis* (GL) and *soleus* (So). *Peroneus* muscles (Pe) are also involved in most of the patients (U). Later in the progression of the disease *tibialis anterior* (TA) and *posterior* (TP) become transformed by fat (V–W).

Cranial involvement was analysed in 73 patients and fatty replacement was detected in 25 (34.2%). The tongue (34.2%) and the cervical paraspinal muscles (24.6%) were most commonly involved (figure 1A–D). In contrast, *temporalis, masseter* and *sternocleidomastoideus* muscles were least commonly affected (only in 1, 2 and 3 patients, respectively). The degree of fat replacement of the cervical paraspinal and *sternocleidomastoideus* muscles had a statistically significant correlation with item 1 ‘lifts head from supine’, from the NSAA-a, although the correlation coefficient was poor (ρ=0.46) (table 1).

**Table 1:** Correlation found between muscle MRI scores and the appropriate muscle function test per every region of the body

Body region	Functional score	Spearman test	Correlation coefficient
Cranial muscles	NSAA item 1 (lift head)	0.0001	−0.46
Arm muscles	Brooke score	0.0001	0.77
	MMT biceps*	0.0001	−0.68
Scapular muscles	Brooke scale	0.0001	0.68
	ACTIVLIM scale	0.0001	−0.57
Trunk/pelvic muscles	NSAA item 12–13 (stand on one leg)	0.0001	−0.74
	NSAA item 6 (get to sitting)	0.0001	−0.72
	MMT glutei†	0.0001	−0.471
Thigh muscles	Time to Climb 4 Steps	0.0001	0.63
	Time to Up & Go	0.0001	0.63
	ACTIVLIM Up Stairs	0.0001	0.53
	ACTIVLIM Down Stairs	0.0001	0.68
	MMT knee flexion‡	0.0001	−0.63
Leg muscles	NSAA item 20 (stand on heels)	>0.05	
	NSAA item 28 (stand on tiptoes)	>0.05	
	MMT plantar flexion§	>0.05	
Trunk, pelvis, thigh muscles	6MWT	0.0001	−0.73
	Time to run/walk 10 m	0.0001	0.55

*Correlation between the degree of muscle fatty transformation of the *biceps brachii* and the MMT of biceps.

†Correlation between mean degree muscle fatty transformation of the glutei muscles and MMT of hip extension.

‡Correlation between the mean degree of muscle fatty transformation of the posterior muscles of the thighs and MMT of knee flexion.

§Correlation between the mean degree of muscle fatty transformation of the posterior muscles of the lower legs and MMT of plantar flexion.

MMT, manual muscle test; MWT, minute walking test; NSAA, North Star Ambulatory Assessment.

### Arm muscles

Arm involvement was analysed in 35 patients. Hierarchical analysis identified two more commonly and severely involved muscles (online supplementary figure 1A): the *biceps brachii* (57.1%) and the anterior muscles of the forearm (53.8%). Commonly observed patterns included:

1. The *biceps brachii* was equally or more severely involved than the *triceps* or the *brachialis* muscles (figure 1I,J, online supplementary figure 1 and table 2).
2. The anterior muscles of the forearm were equally or more severely involved than the posterior muscles of the forearm (figure 1K and online supplementary figure 1).

10.1136/jnnp-2017-317488.supp4 Supplementary file 4

**Table 2:** Percentage of patients for whom the every ‘pattern rule’ proposed was correct

Criteria	% of cases
*Biceps brachii* equally or more severely involved than the *triceps*	100
*Biceps brachii* equally or more severely involved than *brachialis*	97.05
Anterior muscles of the forearm more severely involved than posterior muscles of the forearm	95.05
Patients in which s*ubscapularis* was involved despite not having symptoms of proximal upper limb muscle dysfunction as measured by the Brooke test and the manual muscle testing	67.64
The *supraspinatus* was equally or more severely involved than the *rhomboideus*	97.01
The *infraspinatus* was equally or more severely involved than the *rhomboideus*	98.38
The *latissimus dorsi* was equally or more severely involved than the *serratus anterior*	95.55
If *levator scapulae* and *rhomboideus* were affected, *subscapularis, supraspinatus* and *infraspinatus* were severely involved	77.77
Paraspinal muscles were equally or more involved than abdominal muscles	95.4
*Gluteus minimus* equally or more severely involved than *gluteus medius*	98.86
*Gluteus minimus* equally or more severely involved than *gluteus maximus*	95.45
*Obturator externus* equally or more severely involved than the *gluteus maximus*	89.87
*Biceps femoris long head* equally or more involved than the *biceps femoris short head*	95.18
*Adductor magnus* equally or more severely involved than the *adductor longus*	80.98
*Rectus femoris* (Score1 to 4) was not spared when the *vasti* muscles were involved (Score 2, 3 or 4)	92.61
S*artorius* and *gracilis* were not completely replaced by fat	92.22
All symptomatic patients had involvement of at least one posterior muscle in the lower legs	100
*Peroneus* equally or more involved than the *tibialis anterior*	91.57

### Scapular muscles

**Figure 2:**
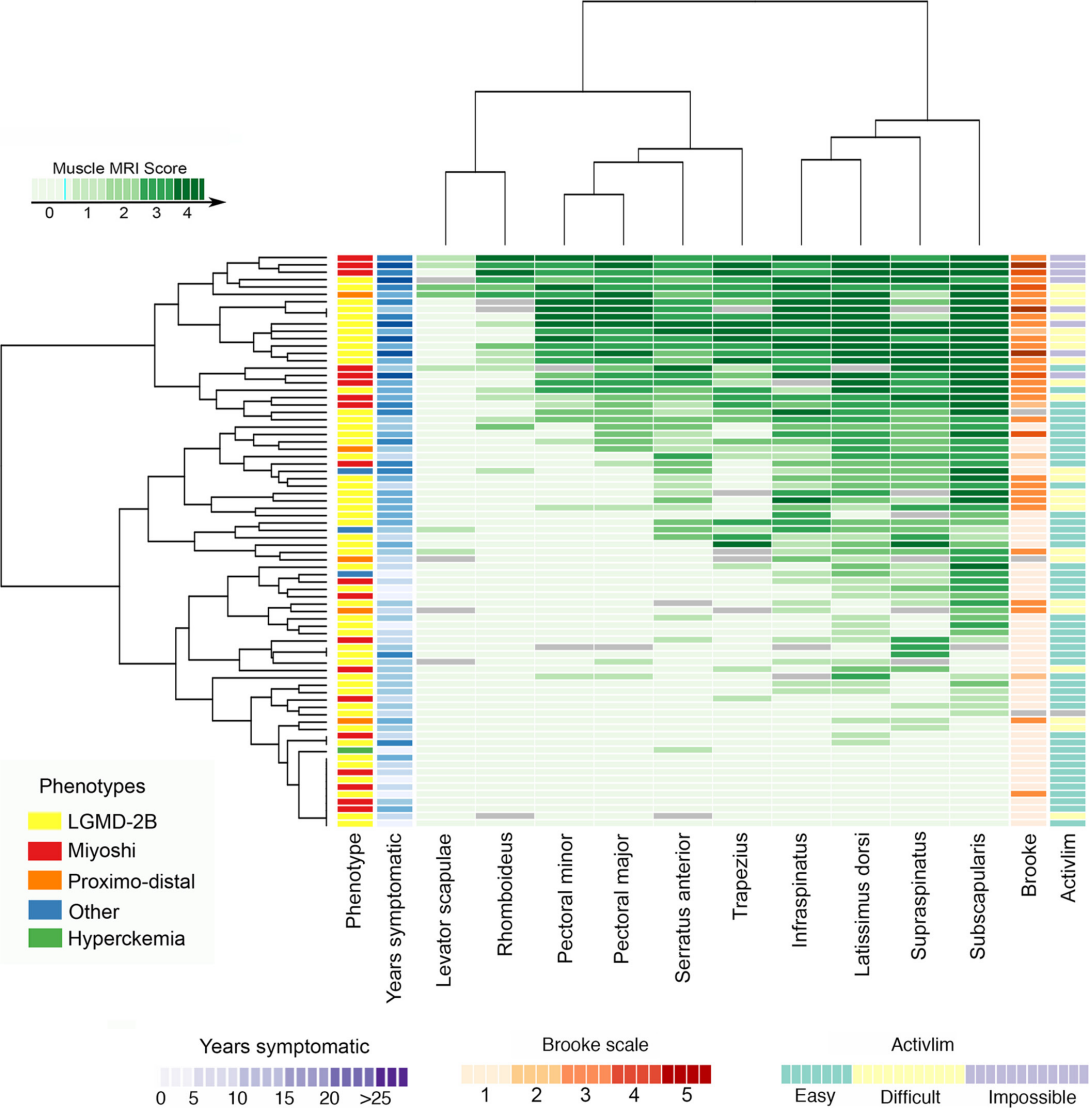
Heatmaps showing involvement of scapular muscles. Patients and muscles are ordered according to hierarchical clustering with increasing grading in fat replacement severity from the bottom to the top (patients—rows) and from the left to the right (muscles—columns). The score of a muscle in a patient is indicated by the colour of the square. Grey squares mean that data are not available. The column in the top left contains information related to the phenotype of the patient at onset of the disease (legend in the bottom left). We have also included a column with information about the time from onset of symptoms to the MRI (years symptomatic) in blue and a column to the far right with the results of the Brooke and ACTIVLIM scales (see legends for these scales at the bottom of the figure): the darker the square, the more time from onset (blue) or the worse the result of the Brooke (orange) or ACTIVLIM scales. We found a statistically significant correlation between the median value of the Mercuri score per patient, the years symptomatic and the results of the Brooke and ACTIVLIM scale. LGMD-2B, limb girdle muscular dystrophy type 2B.

Scapular involvement was analysed in 78 patients. Hierarchical analysis identified four muscles as more commonly and severely involved (figure 2): the *subscapularis* (80.8%), *latissimus dorsi* (75.3%), *infraspinatus* (73.8%) and *supraspinatus* (72.8%).

Commonly observed patterns included:

1. The s*ubscapularis* could be involved in patients even without symptoms of proximal upper limb muscle dysfunction as measured by the Brooke or MMT (figure 1E and figure 2).
2. The *supraspinatus* and *infraspinatus* were equally or more severely involved than the *rhomboideus* (figure 1F–H and figure 2).
3. The *latissimus dorsi* was equally or more severely involved than the *serratus anterior* (figure 1I,J and figure 2).
4. If *levator scapulae* and *rhomboideus* were affected, *subscapularis, supraspinatus* and *infraspinatus* were severely involved (Mercuri 3–4) (figure 1C–H and figure 2).

### Trunk and pelvic muscles

Trunk and pelvic involvement was analysed in 89 patients. Hierarchical analysis identified six more commonly and more severely involved muscles (online supplementary figure 2): the *tensor fascia latae* (95%), *gluteus minimus* (90.8%), *obturator externus* (86%), *iliocostalis* (93.1%), *longissimus* (86.2%) and *multifidus* (88.5%).

10.1136/jnnp-2017-317488.supp5 Supplementary file 5

Commonly observed patterns included:

1. Paraspinal muscles were equally or more involved than abdominal muscles (figure 1J,K and online supplementary figure 2).
2. The *gluteus minimus* was more severely involved than *gluteus medius* and *gluteus maximus* (figure 1L,M and online supplementary figure 2).
3. The *obturator externus* was equally or more severely involved than the *gluteus maximus* (figure 1L–O and online supplementary figure 2).

### Thigh muscles

**Figure 3:**
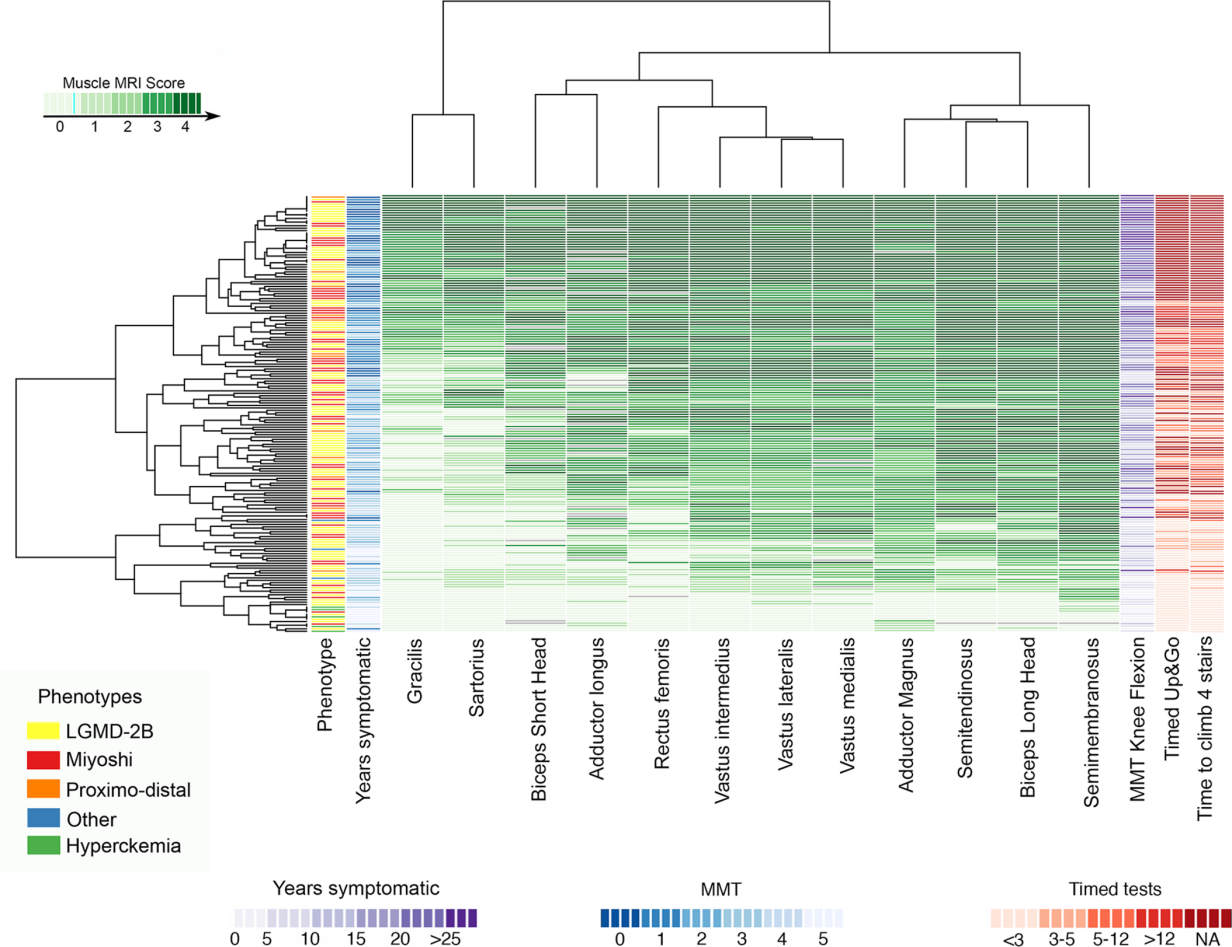
Heatmap of the muscle involvement of the thigh muscles. Patients and muscles are ordered according to hierarchical clustering with increasing grading in severity of fat replacement from the bottom to the top (patients—rows) and from the left to the right (muscles—columns). The score of a muscle in a patient is indicated by the colour of the square. Grey squares means that data is not available. A column in the far left contains information related to the phenotype of the patient at onset of the disease (Yellow: LGMD-2B; red: Miyoshi). We have also included a column with information about the time from onset of symptoms to the MRI (years symptomatic) in blue and a column on the far right with the results of the Timed Up & Go and Time to Climb 4 Stairs tests in red: the darker the square the more time from onset (blue) or the worse the result of the Time to Up & Go (red). We found a statistically significant correlation between the median value of the Mercuri score per patient, the years symptomatic and the results of the time to Up & Go test. LGMD-2B, limb girdle muscular dystrophy type 2; MMT, manual muscle testing.

Thigh involvement was analysed in 182 patients. Hierarchical analysis identified four muscles that were more commonly and more severely involved (figure 3): the *semimembranosus* (95.4%), *semitendinosus* (90.2%), *biceps femoris long head* (93.5%) and *adductor magnus* (94.1%).

Commonly observed patterns included:

1. The *biceps femoris long head* was equally or more involved than the *biceps femoris short head* (figure 1P–S and figure 3).
2. The *adductor magnus* equally or more severely involved than the *adductor longus* (figure 1P–S and figure 3).
3. *Rectus femoris* was not spared when the *vasti* muscles were involved (Mercuri 2, 3 or 4) (figure 1P–S and figure 3).
4. The *sartorius* and *gracilis* were not involved until late stages, but even then were not commonly completely replaced by fat (figure 1P–S and figure 3).

### Lower leg muscles

**Figure 4:**
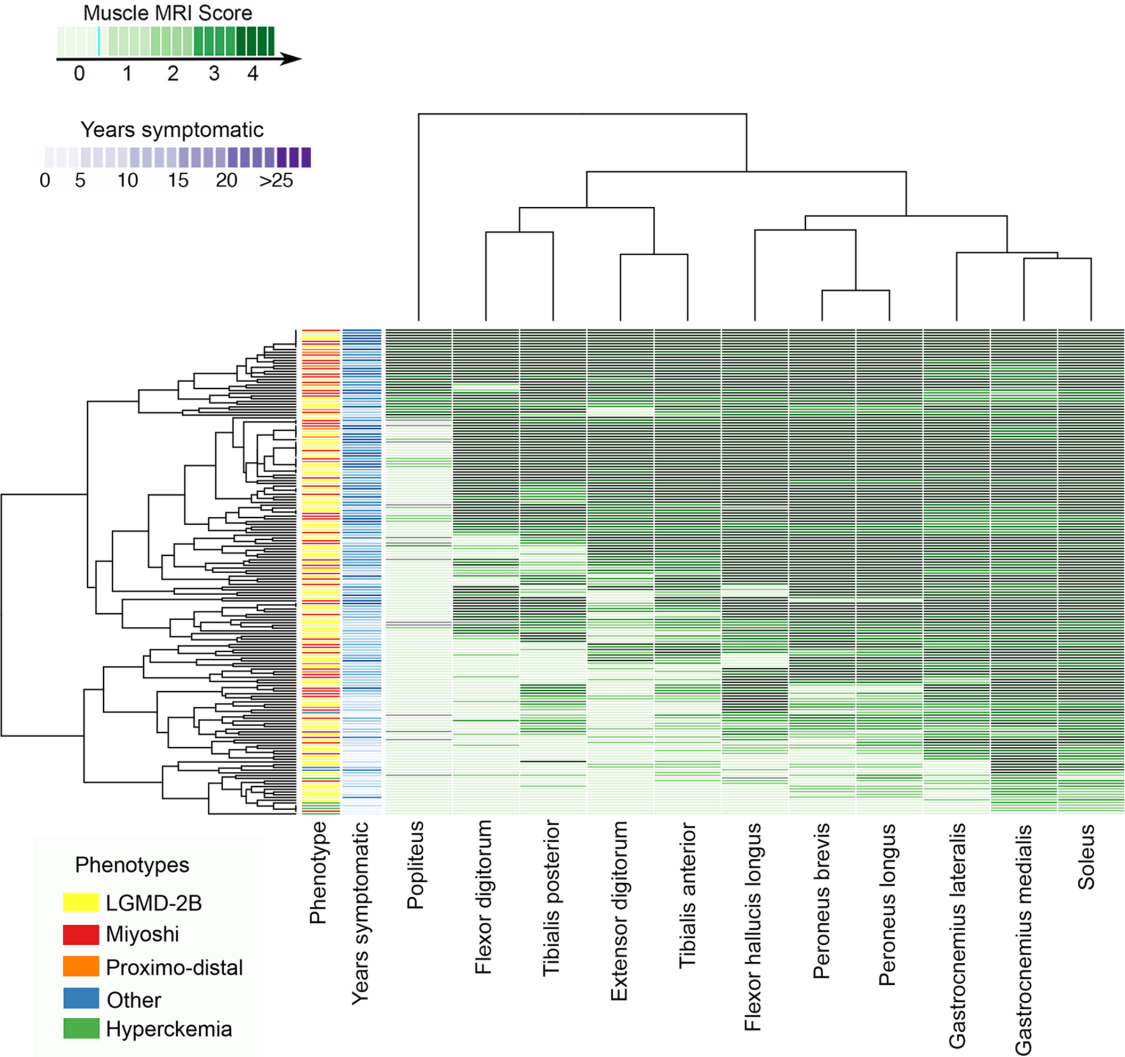
Heatmap of the muscle involvement of the lower leg muscles. Patients and muscles are ordered according to hierarchical clustering with increasing grading in fat replacement severity from the bottom to the top (patients—rows) and from the left to the right (muscles—columns). The score of a muscle in a patient is indicated by the colour of the square. Grey squares means that data is not available. A column in the far left contains information related to the phenotype of the patient at onset of the disease (Yellow: LGMD-2B; red: Miyoshi). We have also included a column with information about the time from onset of symptoms to the MRI (years symptomatic) in blue. We found a statistically significant correlation between the median value of the Mercuri score per patient and the years symptomatic. LGMD-2B, limb girdle muscular dystrophy type 2B.

Lower leg involvement was analysed in 182 patients. Hierarchical analysis identified three more commonly and more severely involved muscles (figure 4C): the *soleus* (99.45%), *gastrocnemius medialis* (99.45%) and the *gastrocnemius lateralis* (94.7%).

Commonly observed patterns included:

1. All symptomatic patients had involvement of at least one posterior muscle of the lower legs (figure 1T–W and figure 4).
2. *Peroneus* equally or more involved than the *tibialis anterior* (figure 1T–W and figure 4).
3. Patients with advanced disease could have involvement of all lower leg muscles, including the *tibialis posterior* (figure 1T–W and figure 4).

### Influence of demographic and clinical features on muscle involvement

There were gender differences in the degree of muscle fatty involvement in some muscles. The *rectus abdominis, rectus femoris, vastus intermedius, vastus lateralis, vastus medialis, peroneus longus, peroneus brevis, gastrocnemius lateralis, gastrocnemius medialis and soleus* were more severely involved in women (Mann Whitney U test, p<0.05). Ethnicity did not appear to influence the degree of involvement. Neither the type of mutation nor the phenotype at onset correlated with the pattern or severity of the fat replacement on MRI.

### Influence of disease duration on the degree of muscle pathology: a natural history approach

**Figure 5:**
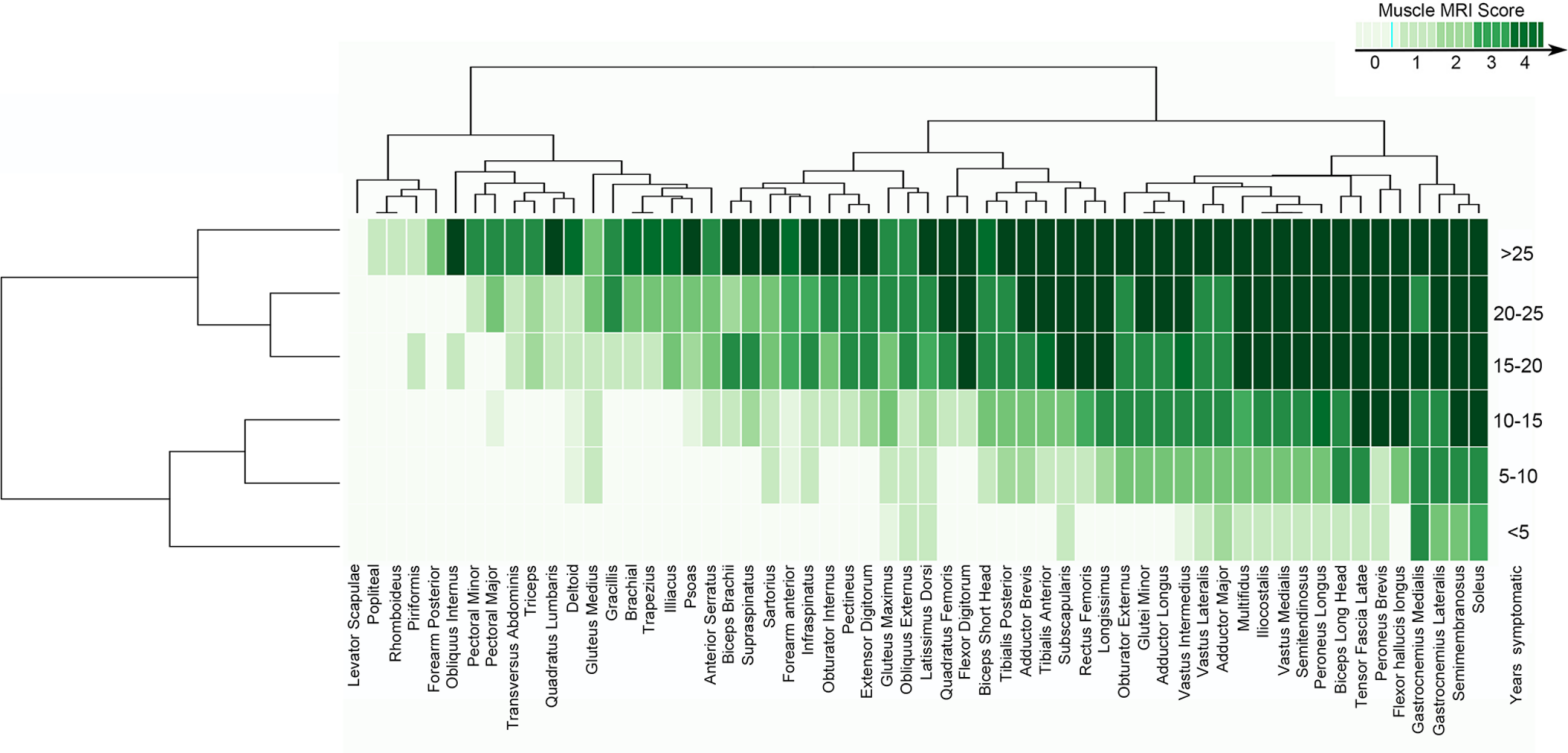
Heatmap showing the progression of the muscle involvement related to the time from onset of symptoms to the MRI. Patients were divided into six groups for the analysis of the progression of muscle involvement. Muscles (columns) are ordered according to hierarchical clustering with increasing grading of muscle fatty replacement in T1-W imaging from the left to the right. The score of a muscle per every group is indicated by the colour of the square. We obtained a pattern of the progression of the disease related to the time from onset of symptoms to the MRI showing the natural history of the disease.

Disease duration (defined here as time since onset of muscle weakness) correlated with the degree of fat replacement in all anatomical regions (Spearman’s test, p<0.0001). To investigate the sequence of muscle involvement as the disease progressed, we classified the patients into six groups depending on the time to MRI from onset and calculated the median value of fat replacement of muscle tissue. The heatmaps obtained revealed a pattern of disease progression (figure 5). The muscles involved in the earliest stages of the disease were predominantly in the lower limbs, pelvis or trunk, although the s*ubscapularis* and the *latissimus dorsi* could also be involved early (figure 5). The rate at which fatty replacement progressed varied between muscles, for example, the *soleu*s, *gastrocnemius medialis* and *semimembranosus* were usually completely involved after 10 years, while the *adductor magnus* or *vastus lateralis* were only completely involved when disease duration approached 25 years.

### Correlation between functional tests and the muscle Mercuri score

The degree of fatty replacement on semiquantitative muscle MRI was correlated with the Brooke scale for the upper limbs, the 6 min walking test (6MWT) for the lower limbs and the NSAA, MMT and timed tests (table 1).

For the scapular muscles, we classified the patients into five groups according to the Brooke scale (the two least able scores were grouped together and indicated by the number 5) and calculated the median value of fat per muscle in every group of patients. The heatmap obtained (online supplementary figure 3) showed that the *subscapularis*, *latissimus dorsi*, *infraspinatus* and *supraspinatus* were involved in all patients, even if they were in the most functional group (ie, score of 1), while the *levator scapulae* or *rhomboideus* were only mildly involved, even in the least able patients (score 4 and 5).

10.1136/jnnp-2017-317488.supp6 Supplementary file 6

**Figure 6:**
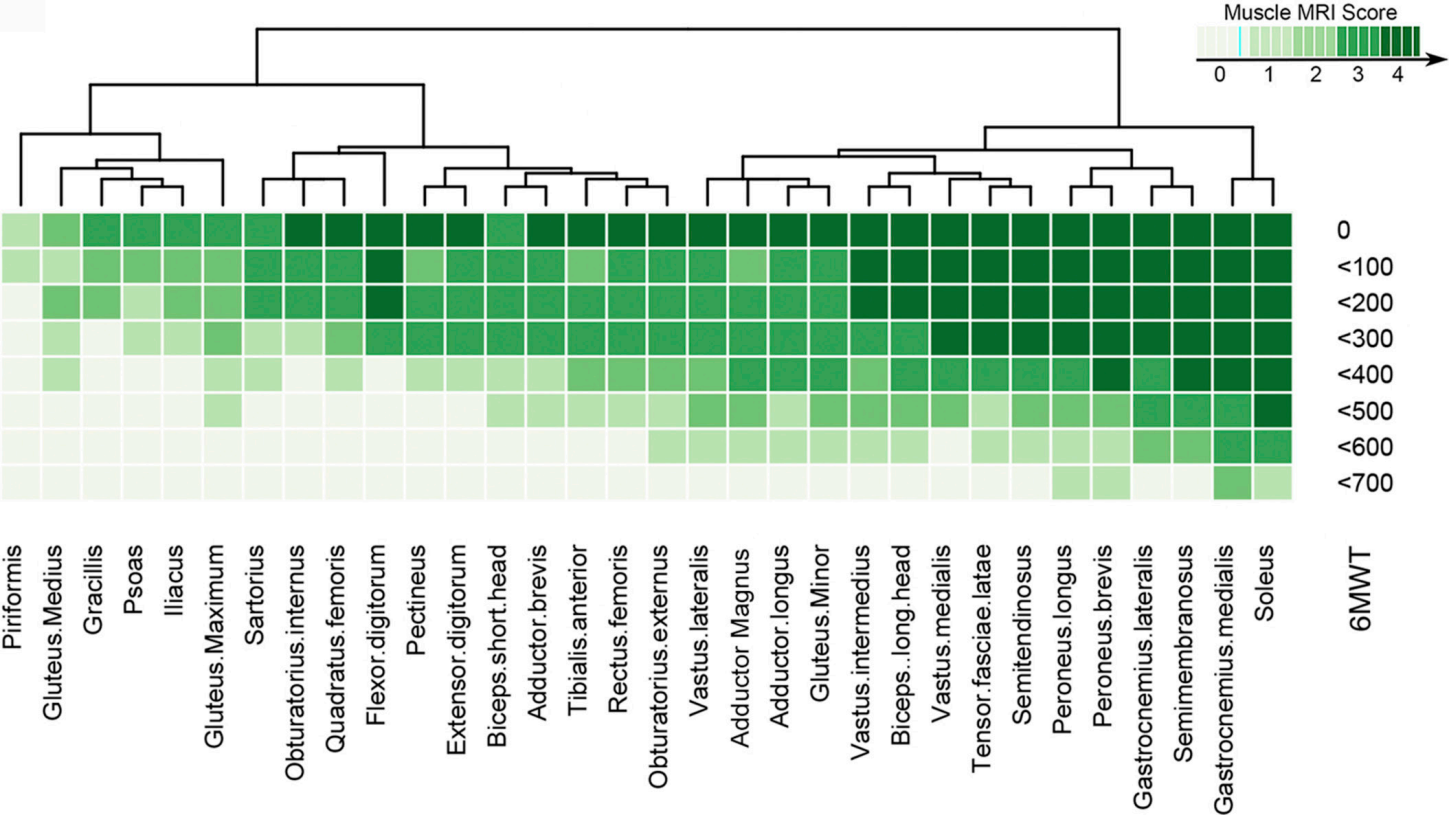
Heatmap showing the progression of the muscle involvement related to the distance covered in the 6MWT. Patients were divided into eight groups depending on the distance covered in the 6MWT for the analysis of the progression of muscle involvement. Muscles (columns) are ordered according to hierarchical clustering with increasing grading of muscle fatty transformation in T1-W imaging from the left to the right. The score of a muscle per group is indicated by the colour of the square. We obtained a pattern of the progression of the disease in muscles of the pelvis, thighs and lower legs related to the functional test 6MWT.

For the lower limbs, we classified patients into groups depending on distance covered in the 6MWT and calculated the median value of fat replacement for each muscle (figure 6). Using this approach, we suggest which muscles would be useful to follow longitudinally with muscle MRI according to the result of the 6MWT. For example, in the case of patients walking 600–700 m, the *soleus*, *gastrocnemius medialis* and the *peroneus* group were already involved, though to a mild degree. In contrast, these four muscles were completely transformed in patients walking 300–400 m and would therefore not be suitable for follow-up once function has decreased to this level. At this point, the graphic shows that the *tibialis anterior*, *vastus intermedius* or even the *vastus medialis* would be more useful for follow-up. In non-ambulant patients, the muscles useful for follow-up included the *gracilis*, the *gluteus medius* or the *piriformis*.

## Discussion

We have characterised muscle involvement on MRI in a large and clinically heterogeneous cohort of patients with dysferlinopathy. This large cross-sectional cohort has permitted better characterisation of the pattern of muscle involvement in dysferlinopathy and improved our understanding of disease progression. This information will support both diagnostics and clinical trial development.

We have defined a characteristic pattern of muscle involvement for dysferlinopathy, independent of the clinical phenotype. An early combination of fat replacement in distal posterior muscles of the lower limbs (*gastrocnemius medialis* and *soleus*) together with involvement of posterior muscles of the thighs (*semimembranosus, semitendinosus, adductor major*), pelvic muscles (*tensor fascia latae* and *obturator externus*), paraspinal muscles (*multifidus, iliocostalis*) and the scapular muscles (*subscapularis* and *latissimus dorsi*) can be helpful for differential diagnosis of patients with a muscle dystrophy and lead to an early diagnosis of dysferlinopathy. Moreover, some muscles are not involved until very late in the disease such as the *levator scapulae*, the *rhomboideus* the posterior muscles of the forearms, the *gluteus maximus* and *medius* muscles and the *gracilis*. This pattern of muscle involvement and sparing could be helpful for differential diagnosis by MRI in severely affected patients, in whom obtaining a muscle biopsy may be challenging.

We confirmed that there are no differences in the pattern of muscle involvement between patients with LGMD-2B, MM or other phenotypic presentations.^9–11^ This suggests that these subtypes of dysferlinopathy are not truly different and that a unifying pathophysiology is likely, similar to findings in other muscle diseases, such as the laminopathies, where different mutations result in similar patterns of selective muscle pathology.^17^ Extensive MRI investigations, rather than clinical descriptions, are therefore more likely to be helpful in understanding the pathophysiology of disease or the role of genetic modifiers. As previously mentioned, some muscles were more involved in women than men. Although we do not have a clear explanation to explain this issue, it is well known that testosterone and estrogens influence skeletal muscle homeostasis and metabolism and probably have an effect on muscle wasting in muscle dystrophies.^18^ Exercise has been shown to be important for muscle size and metabolism, which may be a factor rather than gender here.

The pattern of muscle involvement reported here can be considered characteristic for dysferlinopathy and contributes to differential diagnosis between other muscle diseases with limb girdle weakness. Patients with Becker muscular dystrophy tend to have early fatty replacement of the *glutei medius* and *maximus* while in contrast the *adductor longus* tends to be preserved until later stages.^18 19^ Muscle MRI scans of patients with LGMD-1B show no involvement of the *vasti* until later stages although the *rectus femoris* tends not to be affected and is often hypertrophic.^17 20^ Patients with LGMD-2A tend to have more severe involvement of the *glutei* and the *vasti* are less involved than the posterior muscles of the thighs.^21 22^ This pattern is similar in patients with LGMD-2I.^23^ Patients with sarcoglycanopathy, in whom there is usually no involvement of muscles of the lower legs before loss of ambulation, are easy to distinguish.^24^ Patients with adult-onset Pompe, which presents frequently with limb girdle weakness, have a muscle MRI pattern characterised by early involvement of axial, abdominal, gluteal and posterior thigh muscles, with the muscles of the lower legs not commonly involved.^25 26^ Patients with mutation in *ANO5* (LGMD-2L) have a similar pattern of muscle involvement compared with patients with dysferlinopathy, with predominant involvement of the posterior muscles of the lower legs associated with involvement of posterior and anterior muscles of the thighs without involvement of the *glutei.* 27–29 However, some differences can be found: the *gluteus minimus* and *medius* seem more commonly involved in LGMD-2L than in LGMD-2B and asymmetric involvement is more pronounced in LGMD-2L than dysferlinopathy.^26 28^

Previous similar radiological studies in dysferlinopathy have been performed in lower limbs only and involved small cohorts.^9–11 30^ Although these studies describe some of the features found here, we have expanded the range of patients analysed, using data from patients of different ethnic origins at many different stages of the disease.

We presented the pathology scores from T1-weighted images as heatmaps, as has been recently described for other muscle disorders.^31 32^ This is a new approach to display large amount of data, which simplifies the analysis of several variables. Following this method, we identified a clear correlation between disease duration and the degree of muscle involvement. This analysis raises interesting questions about factors affecting the rate of muscle degeneration. We showed that some muscles were involved early, while others were not involved until much later. In addition, while a group of muscles could become involved at the same time, the progression of fat replacement between muscles in a group could also vary. For example, in most patients, the *gastrocnemius medialis* becomes completely replaced by fat in less than 10 years from symptom onset, while the *biceps femoris long head*, while also involved in the first 10 years, remains minimally affected for longer. It is tempting to hypothesise that muscles with a slower progression could express proteins that protect them from rapid degeneration. Despite previous attempts to investigate this in dysferlinopathy, it is still not clear why muscle degeneration shows a different rate of progression.^33^ The clear understanding of the pattern of severely involved and spared muscles demonstrated here should allow for more focused investigations in the future.

T1-weighted muscle MRI has traditionally been used for differential diagnosis of muscle diseases or to select a suitable muscle to biopsy.^34 35^ As the development of novel therapeutics for many muscular dystrophies progresses, there is a growing need for reliable biomarkers to follow-up patients.^36^ We have shown that muscle MRI findings correlate with the results of most functional tests that may be included in clinical trials (table 1), adding weight to its use as a biomarker. However, as dysferlinopathy is a slowly progressive disorder, changes from 1 year to the next are probably not significant enough to be detectable using semiquantitative T1w imaging. Quantitative sequences such as 3-point Dixon or T2-mapping should be more useful for patient follow-up in short-term longitudinal studies.^37–39^ However, the present study can inform longitudinal quantitative MRI studies regards which muscles to monitor at different stages of disease.

This work is the result of a large international collaboration between different clinical and radiological groups to harmonise MRI protocols. However, although the study includes a high number of patients with different phenotypes and different disease stages, it has some obvious limitations. First, not all patients were studied with whole body muscle MRI due to technical limitations in some centres. Second, the MRI systems used were different, which can include some variability in the data obtained. Third, there are no data regarding **Short-TI Inversion Recovery (**STIR) results, but T2 imaging has been carried out and scans are currently under analysis.

In summary, our study provides information about the distribution and degree of fat replacement of muscle tissue in the largest cohort of patients with dysferlinopathy analysed to date. The study has expanded the characterisation of patterns that can be found in patients with dysferlinopathy, regardless of their clinical phenotype. We have also shown a correlation between muscle pathology as detected by MRI with disease duration and the results of related functional tests, which will inform the design of future clinical trials.
