# Supplementary material for: Muscle MRI in patients with dysferlinopathy: pattern recognition and implications for clinical trials
Source: J Neurol Neurosurg Psychiatry. 2018 May 7;89(10):1071–81. doi: 10.1136/jnnp-2017-317488 (PMC6166612; doi:10.1136/jnnp-2017-317488)
Supplement: Supplementary file 1 [file jnnp-2017-317488supp001.pdf]

__**Supplementary Material 1**__

Idforpub	Phenotype	Mutations 1	Mutations 2	time to walk 10 meters	6MWT
		italics homozygous			
1	LGMD2B	frameshift			
2	LGMD2B	frameshift		8.7	318
3	hyperCKemia	nonsense		2.9	650
4	hyperCKemia	frameshift		2.8	
5	Proximo-distal form of dysferlinopathy	nonsense			
6	hyperCKemia	missense		2.5	633
7	Proximo-distal form of dysferlinopathy	splice site		10.5	258
8	LGMD2B	nonsense		14.9	274
9	Miyoshi myopathy	frameshift		9	355
10	Miyoshi myopathy	frameshift		6	358
11	LGMD2B	frameshift			
13	LGMD2B	frameshift			
14	Miyoshi myopathy	splice site			
15	Proximo-distal form of dysferlinopathy	nonsense		9.2	244
16	LGMD2B	frameshift		12.1	220
17	LGMD2B	splice site		5.2	505
18	LGMD2B	frameshift		9.2	275
19	Miyoshi myopathy	nonsense		9.2	326

Ambulant Status	
**1**	ambulant
**2**	non ambulant

Ethnicity	
**1**	White/ Caucasian
**2**	Asian (Indian Subcontinent)
**3**	Asian (other)
**4**	Black
**5**	Hispanic
**6**	Native indigenous
**7**	Other

20	Proximo-distal form of dysferlinopathy	nonsense			
21	LGMD2B	splice site		4.9	485
22	LGMD2B	splice site		3.6	515
23	LGMD2B	frameshift			
24	Miyoshi myopathy	missense		8.3	346
25	LGMD2B	nonsense		6.7	515
26	Other	splice site		8.2	394
27	LGMD2B	*missense*	*missense*	5.6	506
28	LGMD2B	*frameshift*	*frameshift*	60	
29	LGMD2B	*frameshift*	*frameshift*	24.3	132
30	LGMD2B	*splice site*	*splice site*		
31	LGMD2B	*frameshift*	*frameshift*	17.1	270
32	LGMD2B	*splice site*	*splice site*	9.6	170
33	Proximo-distal form of dysferlinopathy	*missense*	*missense*	10.6	333
34	Proximo-distal form of dysferlinopathy	*missense*	*missense*	41	39
35	LGMD2B	*missense*	*missense*		
37	LGMD2B	*frameshift*	*frameshift*	9.3	325
38	Miyoshi myopathy	*missense*	*missense*		
39	Miyoshi myopathy	*missense*	*missense*		
40	Miyoshi myopathy	*frameshift*	*frameshift*	20.9	150
41	Miyoshi myopathy	*frameshift*	*frameshift*	9	315
42	LGMD2B	*inframe deletion*	*inframe deletion*	19.3	171
43	Miyoshi myopathy	*missense*	*missense*	12.8	241
44	Miyoshi myopathy	*splice site*	*splice site*		
45	Miyoshi myopathy	*nonsense*	*nonsense*	54.3	72
46	Miyoshi myopathy	*frameshift*	*frameshift*	14.2	204

47	LGMD2B	*nonsense*	*nonsense*		
49	LGMD2B	*missense*	*missense*		
50	LGMD2B	*missense*	*missense*		
51	LGMD2B	*frameshift*	*frameshift*	13.5	200
52	LGMD2B	*frameshift*	*frameshift*	3.3	532
53	LGMD2B	*nonsense*	*nonsense*		
54	LGMD2B	*missense*	*missense*		
57	Proximo-distal form of dysferlinopathy	*splice site*	*splice site*	19.4	180
58	LGMD2B	*nonsense*	*nonsense*		
60	LGMD2B	*missense*	*missense*	3.2	641
61	Proximo-distal form of dysferlinopathy	*frameshift*	*frameshift*	11.1	265
63	LGMD2B	*frameshift*	*frameshift*	12.1	
64	LGMD2B	*frameshift*	*frameshift*	14.1	
65	Miyoshi myopathy	*frameshift*	*frameshift*		
66	Miyoshi myopathy	*missense*	*missense*	10.8	285
67	Miyoshi myopathy	*frameshift*	*frameshift*	5.6	475
68	Miyoshi myopathy	*splice site*	*splice site*		
69	LGMD2B	*missense*	*missense*		
70	LGMD2B	*nonsense*	*nonsense*	6.7	390
71	LGMD2B	*nonsense*	*nonsense*	13.3	230
72	Miyoshi myopathy	*frameshift*	*frameshift*	17	
73	Miyoshi myopathy	*frameshift*	*frameshift*	7.3	322
74	Miyoshi myopathy	*frameshift*	*frameshift*	7.7	405
75	Miyoshi myopathy	*missense*	*missense*	20.5	126
76	LGMD2B	*missense*	*missense*	8.8	439
77	Miyoshi myopathy	*nonsense*	*nonsense*		
78	LGMD2B	*splice site*	*splice site*	11.1	260
79	LGMD2B	*frameshift*	*frameshift*	20.3	110

80	LGMD2B	*missense*	*missense*	18.7	150
81	LGMD2B	*missense*	*missense*	8.4	347
82	LGMD2B	*missense*	*missense*	14	
83	LGMD2B	*frameshift*	*frameshift*	7.3	434
84	LGMD2B	frameshift	splice site	21.9	163
85	LGMD2B	splice site	nonsense	27.6	111
86	LGMD2B	frameshift	nonsense		
87	LGMD2B	frameshift	nonsense	5.5	333
88	LGMD2B	splice site	splice site	9.4	225
89	LGMD2B	splice site	frameshift		
90	LGMD2B	missense	nonsense		
91	LGMD2B	nonsense	missense	6.3	468
92	LGMD2B	frameshift	missense	14.8	194
93	Miyoshi myopathy	nonsense	frameshift		
96	Miyoshi myopathy	frameshift	frameshift		
97	LGMD2B	splice site	missense	3.4	618
98	LGMD2B	nonsense	inframe duplication	8.4	365
99	Proximo-distal form of dysferlinopathy	frameshift	missense	15.3	172
100	Proximo-distal form of dysferlinopathy	frameshift	missense	22.8	159
101	LGMD2B	missense	missense	51.2	232
102	LGMD2B	frameshift	frameshift		
103	Miyoshi myopathy	frameshift	frameshift	10.1	267
104	Miyoshi myopathy	missense	frameshift	12.5	235
105	Miyoshi myopathy	frameshift	missense	3.5	444
106	LGMD2B	frameshift	frameshift	18.1	
107	LGMD2B	missense	missense	17.9	185
108	LGMD2B	missense	missense	13.5	275

109	LGMD2B	missense	frameshift	9.4	343
110	Miyoshi myopathy	nonsense	frameshift	51.5	
111	LGMD2B	missense	frameshift		
112	Miyoshi myopathy	nonsense	frameshift	11.8	
113	LGMD2B	missense	frameshift	3.7	499
115	Miyoshi myopathy	nonsense	missense	6.1	410
116	Miyoshi myopathy	nonsense	frameshift	18.9	203
117	LGMD2B	missense	nonsense	6.5	375
117	LGMD2B	missense		7.4	374
118	LGMD2B	missense	nonsense	4	551
119	LGMD2B	nonsense	splice site		
120	Miyoshi myopathy	frameshift	nonsense	8.6	441
121	LGMD2B	frameshift	nonsense	15.3	144
121	LGMD2B	nonsense	frameshift	11	262
122	LGMD2B	frameshift	nonsense	9	315
123	LGMD2B	frameshift	missense	8.3	364
124	Miyoshi myopathy	frameshift	frameshift	6.8	450
125	Miyoshi myopathy	missense	missense	11	285
128	LGMD2B	splice site	frameshift	7.8	408
129	LGMD2B	splice site	splice site		
130	Miyoshi myopathy	splice site	nonsense	7	504
131	LGMD2B	nonsense	frameshift	10.9	288
132	LGMD2B	splice site	nonsense		
133	LGMD2B	frameshift	nonsense		
134	LGMD2B	missense	frameshift		
135	LGMD2B	splice site	frameshift	9.5	314
136	LGMD2B	missense	missense	3.7	567
137	Miyoshi myopathy	frameshift	nonsense	12.2	175
138	LGMD2B	missense	nonsense	10.9	310

139	Proximo-distal form of dysferlinopathy	nonsense	splice site		
140	Miyoshi myopathy	nonsense	frameshift	7.9	403
142	LGMD2B	nonsense	frameshift		
143	Miyoshi myopathy	frameshift	frameshift		
144	Miyoshi myopathy	missense	missense		
145	Miyoshi myopathy	splice site	frameshift	21.6	145
146	LGMD2B	splice site	nonsense		
148	Miyoshi myopathy	frameshift	frameshift	3.2	542
149	LGMD2B	splice site	frameshift	9.4	325
150	LGMD2B	missense	missense	7.9	394
151	LGMD2B	splice site			
153	Miyoshi myopathy	nonsense	splice site	9	392
154	Miyoshi myopathy	splice site	frameshift	10.5	300
155	Other	missense	missense	5.2	331
156	LGMD2B	frameshift	splice site		
157	Miyoshi myopathy	missense	nonsense		
158	LGMD2B	splice site	nonsense	17	
160	LGMD2B	missense	missense	5	500
161	Miyoshi myopathy	frameshift	frameshift	8.6	385
162	LGMD2B	missense	nonsense		
165	Miyoshi myopathy	nonsense	frameshift	8.2	454
166	LGMD2B	nonsense	frameshift		
167	Other	missense	frameshift	11.3	193
168	Proximo-distal form of dysferlinopathy	missense	missense	8.8	321
169	Miyoshi myopathy	missense	frameshift	10	303
170	hyperCKemia	frameshift	splice site	2.8	568
171	Miyoshi myopathy	nonsense	splice site	6.8	447
173	Miyoshi myopathy	frameshift	nonsense	3.3	452

174	Miyoshi myopathy	frameshift	missense	17	150
175	hyperCKemia	nonsense	frameshift	6.3	385
176	Miyoshi myopathy	frameshift	inframe deletion		
177	LGMD2B	frameshift	nonsense		
178	Miyoshi myopathy	nonsense	splice site		
179	Miyoshi myopathy	frameshift	missense	16.8	210
180	LGMD2B	nonsense	missense	4.7	505
182	LGMD2B	frameshift	frameshift	3.5	611
183	LGMD2B	frameshift	frameshift	4.1	
184	LGMD2B	frameshift	frameshift	2.8	636
185	Miyoshi myopathy	frameshift	missense		
186	LGMD2B	nonsense	splice site	13.9	345
187	LGMD2B	missense	nonsense	7.2	392
188	LGMD2B	nonsense	nonsense	7.7	455
189	Miyoshi myopathy	frameshift	missense	3.4	573
190	LGMD2B	splice site	nonsense	8.2	394
191	LGMD2B	splice site	missense	3.3	558
192	LGMD2B	missense	missense	16.5	315
193	LGMD2B	missense	missense	6.7	370
194	LGMD2B	nonsense	nonsense	6.7	400
195	LGMD2B	frameshift			263
196	LGMD2B	frameshift			
197	Miyoshi myopathy	nonsense	missense	7.3	410
198	Miyoshi myopathy	missense			
199	LGMD2B	nonsense	nonsense	13	257
200	LGMD2B	nonsense		5.1	521
