# Supplementary material for: Muscle MRI in patients with dysferlinopathy: pattern recognition and implications for clinical trials
Source: J Neurol Neurosurg Psychiatry. 2018 May 7;89(10):1071–81. doi: 10.1136/jnnp-2017-317488 (PMC6166612; doi:10.1136/jnnp-2017-317488)
Supplement: Supplementary file 2 [file jnnp-2017-317488supp002.pdf]

Supplemental material 2

T1 weighted imaging from whole body or lower limbs were obtained using the following equipment:

Site	Scanner Make	Model	Teslas
Columbus	GE	HDx	3
Sydney	GE	Sigma HDxT	3
Stanford	GE	750	3
Munich	Philips	Achieva	3
Newcastle	Philips	Achieva	3
Paris	Siemens	Magnetom Trio	3
Tokyo	Siemens	Syngo	3
Charlotte	GE	450w	1.5
Washington	GE	MR450W	1.5
Barcelona	Philips	AcievaXR	1.5
Seville	Philips	Achieva	1.5
St Louis	Siemens	VB17	1.5
Padova	Siemens	Avanto VB17	1.5
Marseilles	Siemens	Avanto	1.5

Acquisition Paramaeters

Axial T1 weighted turbo spin echo images were obtained using the following acquisition parameters TR = 757 ms, TE = 17 ms, thickness = 8 mm, number of slices = 164, FOV = 530 x 530 mm, acquired voxel size = 1.6 x 2.88 mm.

The time to obtain all the images was 20 minutes per patient.

MRI analysis

The semiquantitative Mercuri visual scale modified by Fisher was used to score the T1W scan[16]:

0: Normal appearance;

1: Mild involvement: Traces of increased signal intensity on the T1-weighted MR sequences

2: Moderate involvement: increased signal intensity with beginning confluence in less than 50% of the muscle.

3: Severe involvement: increased signal intensity in more than 50% of an examined muscle

4: End stage: when the entire muscle is replaced increased signal intensity.
