# Supplementary material for: Muscle MRI in patients with dysferlinopathy: pattern recognition and implications for clinical trials
Source: J Neurol Neurosurg Psychiatry. 2018 May 7;89(10):1071–81. doi: 10.1136/jnnp-2017-317488 (PMC6166612; doi:10.1136/jnnp-2017-317488)
Supplement: Supplementary file 3 [file jnnp-2017-317488supp003.pdf]

Summary Demographic Data for the 182 patients included in T1W MRI study

Gender	Percentage	Number
Male	48%	94
Female	52%	88

Ethnicity	Percentage	Number
Caucasian	69%	127
Asian- Indian Subcontinent	7%	13
Asian- Other	9%	16
Black	2%	4
Hispanic	8%	15
Native Indigenous	0%	0
Other	4%	7

	Mean	Range
**Age at MRI (years)**	37.59 years	10-86 years
**Years Symptomatic**	15.81 years	0-50 years

	Percentage	Number
**Ambulant**	75.27%	137
**Non ambulant**	24.73%	45

Phenotype	Number
LGMD2B	105
Miyoshi Myopathy	57
HyperCKemia	5
Proximo Distal form of Dysferlinopathy	12
Other	3
