# Supplementary figures and images for: Muscle MRI in patients with dysferlinopathy: pattern recognition and implications for clinical trials
Source: J Neurol Neurosurg Psychiatry. 2018 May 7;89(10):1071–81. doi: 10.1136/jnnp-2017-317488 (PMC6166612; doi:10.1136/jnnp-2017-317488)

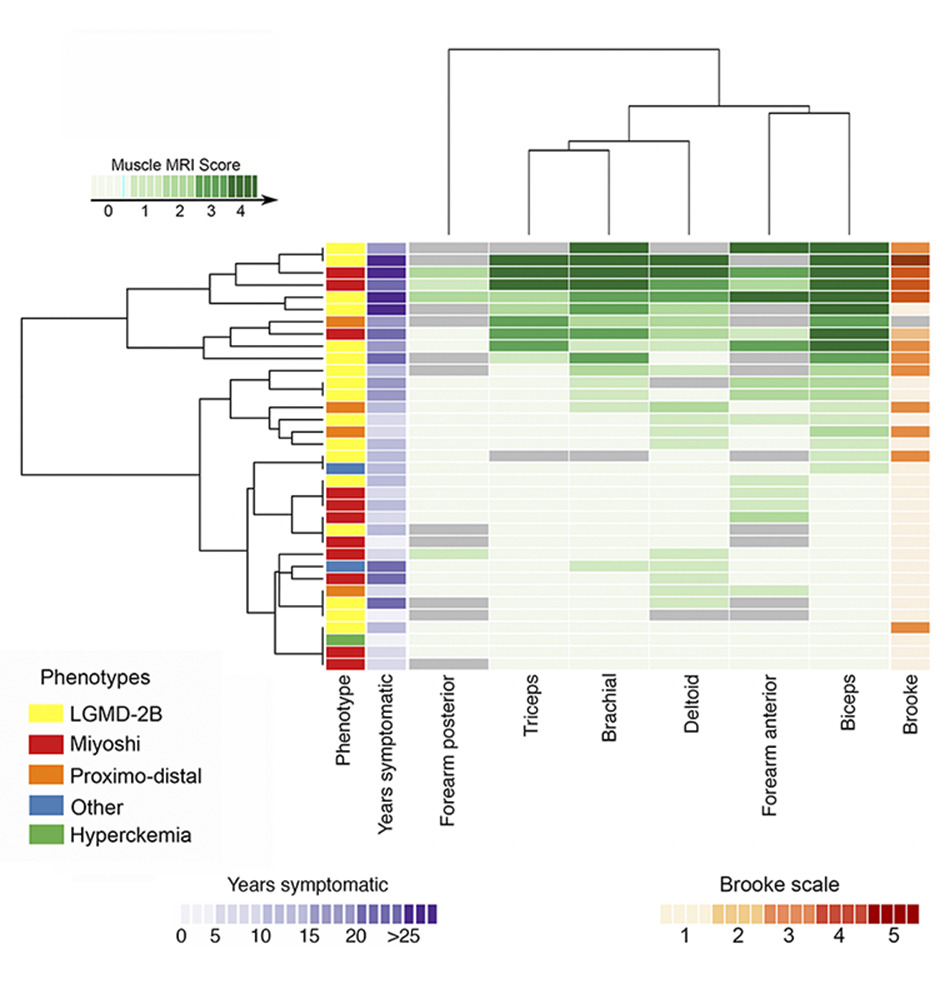

Supplement: Supplementary file 4 [file jnnp-2017-317488supp004.jpg]

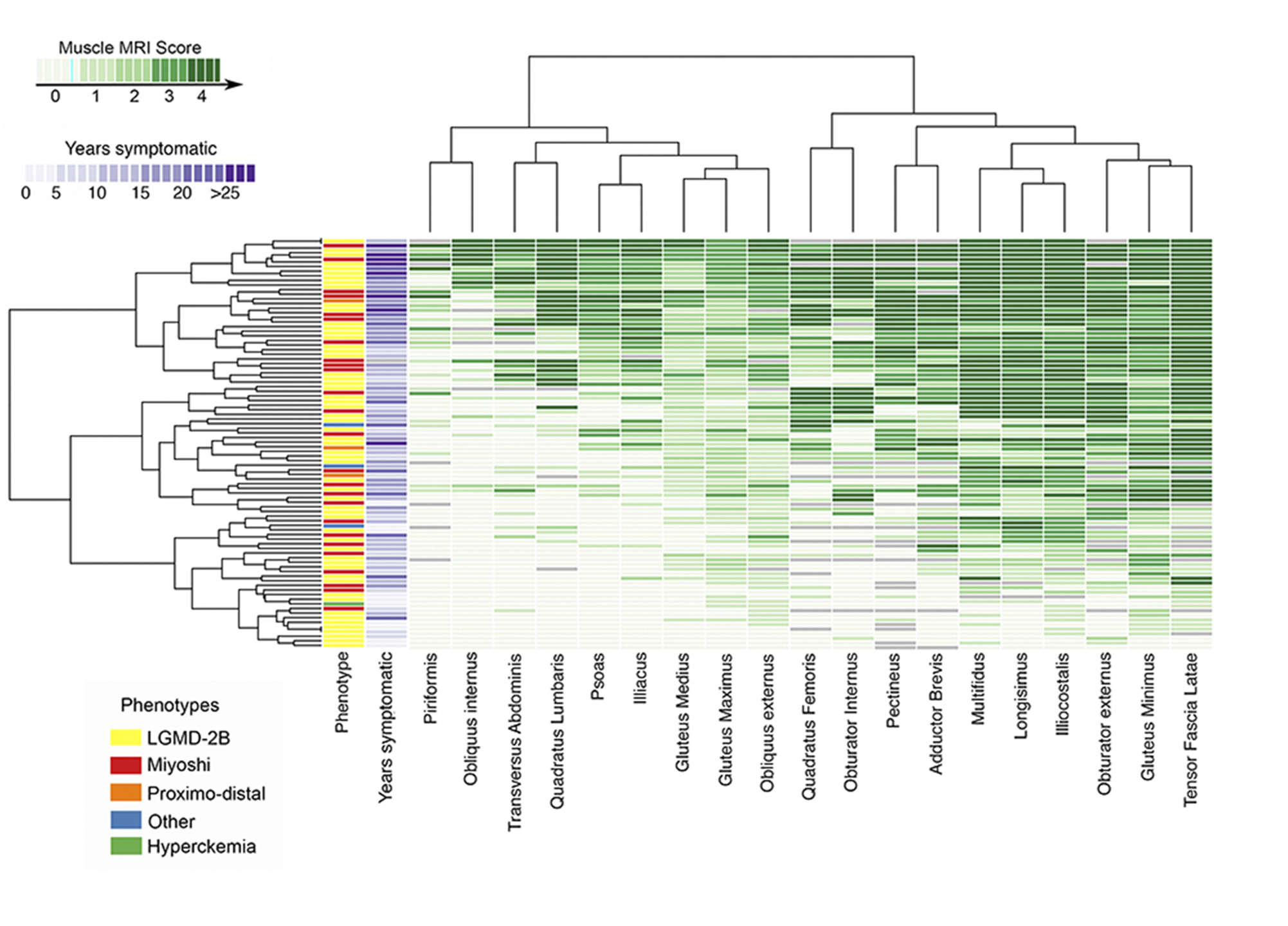

Supplement: Supplementary file 5 [file jnnp-2017-317488supp005.jpg]

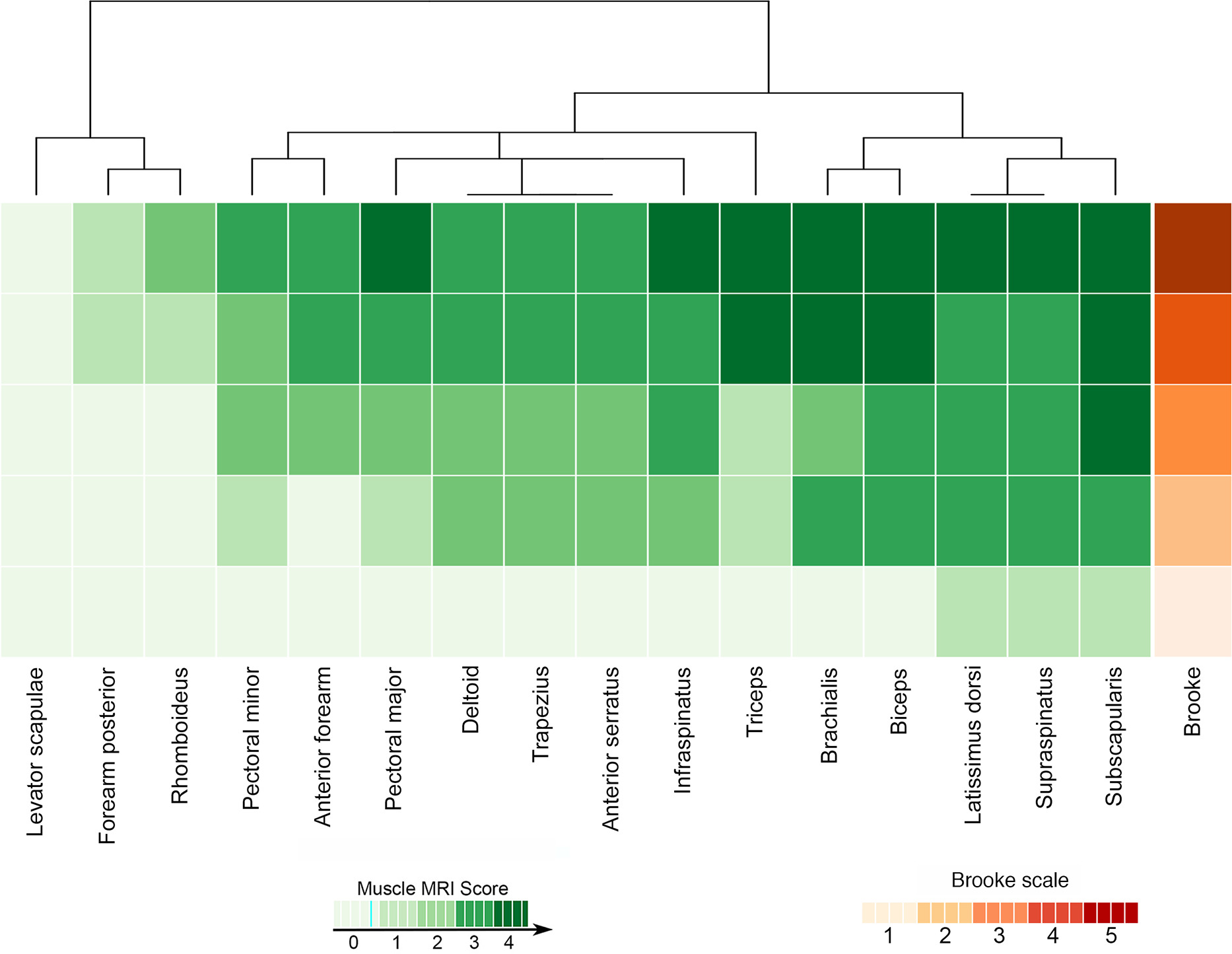

Supplement: Supplementary file 6 [file jnnp-2017-317488supp006.jpg]
